# Effective Antidepressant Chronotherapeutics (Sleep Deprivation and Light Therapy) Normalize the IL-1β:IL-1ra Ratio in Bipolar Depression

**DOI:** 10.3389/fphys.2021.740686

**Published:** 2021-09-01

**Authors:** Francesco Benedetti, Sara Dallaspezia, Elisa Maria Teresa Melloni, Cristina Lorenzi, Raffaella Zanardi, Barbara Barbini, Cristina Colombo

**Affiliations:** ^1^Vita-Salute San Raffaele University, Milan, Italy; ^2^Psychiatry and Clinical Psychobiology, Division of Neuroscience, Scientific Institute IRCCS Ospedale San Raffaele, Milan, Italy; ^3^Mood Disorders Unit, IRCCS Scientific Institute Ospedale San Raffaele, Milan, Italy

**Keywords:** chronotherapeutics, sleep deprivation, light therapy, inflammation, interleukin-1β, antidepressant

## Abstract

**Background:**

Mood disorders associate with peripheral markers of low-grade inflammation, among which circulating levels of interleukin-1β (IL-1β) consistently predict diagnosis and poor outcomes. Antidepressant chronotherapeutics (total sleep deprivation and light therapy, TSD+LT) prompts response in drug-resistant bipolar depression, but its effect on peripheral inflammation were never assessed. Here we explored the effects of TSD+LT on IL-1β signaling.

**Methods:**

We studied the ratio between IL-1β and its receptor antagonist (IL-1β:IL1ra) in 33 healthy participants, and in 26 inpatients with a major depressive episode in course of Bipolar Disorder, before and after treatment with three cycles of repeated TSD+LT, interspersed with sleep recovery nights, administered during 1 week. Treatment effects of mood and on IL-1β:IL1ra were analyzed in the context of the Generalized Linear Model (GLM).

**Results:**

At baseline, patients had higher IL-1β, IL1ra, and IL-1β:IL1ra than controls. Treatment significantly decreased IL-1β:IL1ra, by decreasing IL-1β and increasing IL1ra, the effect being proportional to baseline levels and normalizing values. Patients with higher baseline levels showed the highest decrease in IL-1β:IL-1ra, which associated with the immediate antidepressant response at the first cycle; while patients with lower baseline values showed negligible changes in the IL-1β:IL-1ra, unrelated to treatment response.

**Conclusion:**

We observed a parallel change of inflammatory biomarkers and severity of depression after chronotherapeutics, suggesting that a reduction in inflammation associated with depression could contribute to the mechanism of action of TSD+LT, and warranting interest for controlled studies addressing the role of inflammation in the recovery from bipolar depression.

## Introduction

The immune state of patients affected by a depressive episode during major depressive disorder (MDD) or bipolar disorder (BD) is likely to play a role both, in the pathogenesis of depression, and as a determinant of therapy outcome (Benedetti et al., 2020; Branchi et al., 2021). Raised pro-inflammatory cytokines in peripheral blood of patients with mood disorders parallel low-grade inflammation and glutamatergic dysfunction in the brain (Haarman et al., 2016; Haroon et al., 2016; Franzen et al., 2020), and characterize subtypes of depression which are poor responsive to antidepressant drugs (Arteaga-Henriquez et al., 2019; Benedetti et al., 2021), and progress toward gray and white matter pathology (Haroon et al., 2017; Sugimoto et al., 2018; Green et al., 2021) and cognitive impairment (Haroon and Miller, 2017; Misiak et al., 2018). Possibly as a result of this global deterioration of functioning and resulting hopelessness, immune dysfunction was consistently associated with suicidality independent of the underlying psychiatric diagnosis (Steiner et al., 2013).

In depressed patients with BD, we showed that higher levels of peripheral cytokines and chemokynes associate with diagnosis (Poletti et al., 2021b), with gray and white matter microstructure (Benedetti et al., 2016b; Poletti et al., 2019), and with neurocognitive impairment (Poletti et al., 2021a). In respect to antidepressant response, we studied the 1-week combination of chronotherapeutics (repeated total sleep deprivation and light therapy, TSD+LT) and lithium, a model experimental antidepressant treatment, which allows to explore the multi-target neurobiological mechanisms of antidepressant effects at close time points (Gillin et al., 2001; Benedetti and Smeraldi, 2009). Known effects of TSD and LT, which can be associated with its antidepressant effects, include an enhancement in serotonergic, noradrenergic, and dopaminergic neurotransmission; a reduction in NMDA glutamatergic signaling; changes in brain cortico-limbic metabolism; and a phase-advance of the sleep-wake and activity-rest rhythm (Gillin et al., 2001; Adrien, 2002; Benedetti and Smeraldi, 2009; Benedetti, 2012; Wirz-Justice et al., 2013; Geoffroy and Palagini, 2020). Possibly because of these multiple effects, combined TSD+LT can prompt response in more than one half of the treated patients, also targeting suicidality and partially overcoming treatment resistance (Colombo et al., 2000; Benedetti et al., 2005, 2014), and we observed worse antidepressant response with higher baseline peripheral cytokines (Benedetti et al., 2002, 2017). We also showed that a 1-week effective repeated TSD+LT is able to revert the functional neural correlates of depressive psychopathology implicated in the cognitive generation of affect (Benedetti et al., 2007a), to improve effective connectivity in the cortico-limbic system during the processing of negative emotional stimuli (Vai et al., 2015), and also to improve measures of brain white matter microstructure, such as fractional anisotropy of water diffusion (Melloni et al., 2020), the degree of change being associated to clinical response.

If depressive psychopathology, and brain structure and function, is negatively influenced by low-grade inflammation in BD, fostering resistance to treatment, it can be hypothesized that TSD+LT could achieve its powerful clinical and neurobiological effects by reducing it, but this hypothesis has not yet been tested (Wirz-Justice and Benedetti, 2019). A systematic review and meta-analysis showed that sleep disturbances and long sleep duration, but not short sleep and experimental TSD, associate with increased markers of systemic inflammation (Irwin et al., 2016). Sleep affects various immune parameters in humans (Besedovsky et al., 2019), but very few data are available in patients with mood disorders, who uniquely benefit from therapeutic TSD, contrary to the detrimental effects observed in healthy controls (Wirz-Justice and Van den Hoofdakker, 1999). Three clinical trials are available on the topic. A study of transcriptome-wide gene expression revealed a strong effect of antidepressant TSD on gene expression in pathways involved in circadian rhythms, and in cytokine signaling, immune function, and inflammatory response, the effect being higher in responders (Foo et al., 2019). We showed that TSD+LT causes an increase in stem cell factor (SCF), a neurotrophic factor involved in neuron-microglia interactions fostering an anti-inflammatory milieu, proportional to treatment efficacy (Benedetti et al., 2016a). A study in winter seasonal depression suggested that LT can successfully normalize the pro-inflammatory state of depressed patients, by correcting the higher macrophage activity and lower lymphocyte proliferation associated with the depressive episode (Song et al., 2015).

To test the effect of TSD+LT on peripheral inflammatory markers associates with treatment resistant depression, in a proof-of-concept study here we studied Interleukin-1β (IL-1β), which has been consistently associated with non-response to drugs (Arteaga-Henriquez et al., 2019; Benedetti et al., 2021). A key mediator of the inflammatory response, IL1β exists in balance with its receptor antagonist (IL1ra), it is widely expressed in the brain where it can decrease hippocampal neurogenesis also mediating the inhibitory effects of stress and inducing depressive-like symptoms (Yoshimura et al., 2013; Yang et al., 2015), and associates with reduced functional connectivity within the cortico-striatal reward circuitry in unmedicated depressed patients (Gupta et al., 2016). Studies suggest that an increased IL-1β:IL1ra ratio initiates inflammation that has been implicated in human disease, and is higher in patients with multiple sclerosis where it associates with enhanced glutamate-mediated transmission and excitotoxicity in central neurons (Rossi et al., 2012; Mendiola and Cardona, 2018). Here we assessed the peripheral IL-1β:IL1ra ratio before/after antidepressant TSD+LT.

## Materials and Methods

We studied a convenient sample of 26 patients consecutively admitted to the mood disorder unit of San Raffaele Hospital in Milan, Italy, who met the criteria for a major depressive episode without psychotic features in the course of BD type I (DSM-5 criteria). All patients had been referred for hospitalization by the psychiatrists in charge because of severe and/or treatment-resistant depression. Inclusion criteria were: to be willing to participate; absence of other diagnoses on Axis I, pregnancy, history of epilepsy, major medical and neurological disorders; no treatment with long-acting neuroleptic drugs in the last 3 months before admission; absence of a history of drug or alcohol dependency; absence of inflammation related symptoms, including fever and infectious or inflammatory disease; uncontrolled systemic disease; uncontrolled metabolic disease or other significant uncontrolled somatic disorder known to affect mood; somatic medications known to affect mood. Physical examinations, laboratory tests and electrocardiograms were performed at admission. After complete description of the study to the subjects, a written informed consent was obtained. All the research activities were approved by the local ethical committee.

All patients were administered three consecutive TSD cycles, each composed of a 36-h period of wakefulness. On days 0, 2, and 4, patients were totally sleep deprived from 07:00 to 19:00 of the following day. They were then allowed to sleep during the night in the sleep window of 19:00–08:00 on days 1, 3, and 5. Patients were administered LT (exposure to a 10,000 lux white light, color temperature 4,600 K, for 30 min) at 03:00 during the TSD night and in the morning after recovery sleep, 30 min after awakening, between 08:00 and 09:00. TSD took place in the common rooms of the ward, with artificial lighting, with no restrictions to the use of cell phones, tablets, or laptops. We administered light at night at the point of maximum sleepiness, to counteract it and avoid microsleeps (Colombo et al., 2000); and in the morning soon after awakening from the recovery sleep to exploit the phase-advance effects of LT, which have been associated with the clinical effects of combined TSD+LT (Benedetti et al., 2007b). Patients who had been taking lithium (*n* = 12) continued to do so, and those who had not (*n* = 14) started lithium along with the chronotherapeutic procedure to enhance its effect and prevent relapse (Benedetti et al., 2014). No other antidepressants were administered. Severity of depression was rated on days 0 (baseline), 1 (the day after first TSD), 2 (the day after first recovery sleep), and 6 (end of treatment) by the psychiatrists in charge according to a modified version of the 21-item HDRS (HDRS-NOW) (Leibenluft et al., 1993), from which items that could not be meaningfully rated due to the TSD procedure (i.e., weight changes and insomnia: item numbers 4, 5, 6, and 16) were excluded.

Blood sampling for the assessment of IL-1β and IL1ra was performed in the morning of the day before the start of treatment (Day 0), and at the end of the 3 treatment cycles (Day 6). Plasma levels of analytes were measured using a bead-based Luminex system based on xMAP technology (Bio-Rad Laboratory, Hercules, CA, United States). Thirty-three healthy participants, recruited via advertising, served as controls for the baseline levels of IL-1β:IL1ra.

Considering the sample size, and given that distributions in patients before/after treatment and in HC were expected to be non-normal (Arteaga-Henriquez et al., 2019; Benedetti et al., 2021), and to differ in variance but having at the most only a small difference in location (Shields, 2020), IL-1β:IL1ra ratio values were compared between groups with simple non-parametric testing (Wald-Wolfowitz Runs Test and Wilcoxon Matched Pairs Test as appropriate). Homogeneity of variances was tested with Levene’s test. To assess the relationship between changes of IL-1β:IL1ra ratio and clinical antidepressant effect, testing the effect of predictors on outcomes, we then performed a repeated measures ANOVA in the context of the General Linear Model (GLM). Considering moreover the *a priori* expected significant interaction with other independent factors (age, sex) and the violation of parametric assumptions (distribution of variables, homogeneity of variances), independent variables were also entered into a Generalized Linear Model (GLZM) analysis of homogeneity of variances, with an identity link function (McCullagh and Nelder, 1989). Parameter estimates were obtained with iterative re-weighted least squares maximum likelihood procedures. The significance of the effects was calculated with the likelihood ratio (LR) statistic, which provides the most asymptotically efficient test known, by performing sequential tests for the effects in the model of the factors on the dependent variable, at each step adding an additional effect into the model contributing to incremental Chi-square statistic, thus providing a test of the increment in the log-likelihood attributable to each current estimated effect (Dobson, 1990; Agresti, 1996). The quality of the statistical model was checked using the entropy maximization principle of the Akaike information criterion (AIC) (Akaike, 1974). All analyses were performed with a commercially available package and using standard computational procedures (StatSoft Statistica 12 Hill and Lewicki, 2006).

## Results

Clinical and demographic characteristics and IL-1β and IL1ra values of participants are resumed in Table 1. Age and sex distributions did not significantly differ between patients and HC.

**TABLE 1:** Clinical and demographic characteristics of participants, and plasma levels of IL-1β and IL1ra.

	**Patients with BD**		**Healthy controls**
	**Before treatment**	**After treatment**	
Sex (M/F)	8/18		12/21
Age	45.35 ± 12.85		42.21 ± 10.07
Age at onset of illness	28.96 ± 11.13		−
Duration of illness (years)	16.35 ± 12.58		−
Previous depressive episodes (n)	5.36 ± 4.17		−
Previous manic episodes (n)	2.88 ± 2.24		−
Duration of current episode (weeks)	20.72 ± 23.71		−
HDRS-NOW score	20.23 ± 4.04	5.77 ± 4.35	−
IL-1β	1.39 ± 1.81	1.04 ± 0.81	0.43 ± 0.61
IL1ra	170.07 ± 75.03	201.85 ± 83.51	81.08 ± 62.48
IL-1β:IL1ra ratio	0.010646 ± 0.013033	0.005320 ± 0.004104	0.00611 ± 0.004670

**FIGURE 1:**
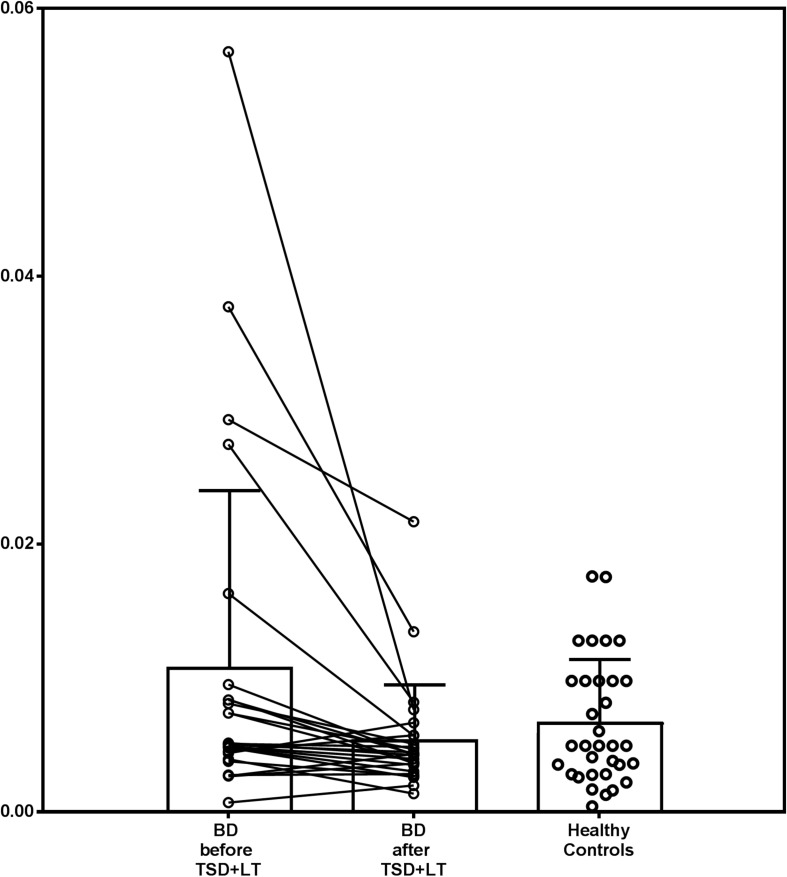
IL1β:IL1ra ratio in patients with bipolar depression treated with chronotherapeutics and in healthy controls.

Baseline IL-1β and IL1ra were significantly higher in patients (adjusted *Z* = 3.620, *p* = 0.0003, and *Z* = 2.821, *p* = 0.0048, respectively). The baseline IL-1β:IL1ra ratio was higher in patients than in HC, too (adjusted *Z* = 2.554, *p* = 0.0106), and with a higher variance (Levene’s *F* = 8.106, *p* = 0.0061). After treatment, IL-1β:IL1ra was comparable in patients and controls (adjusted *Z* = 0.422, *p* = 0.673), with marginal differences in variance (Levene’s *F* = 3.963, *p* = 0.0513). This was because the IL-1β:IL1ra significantly decreased in patients after treatment (adjusted *Z* = 3.213, *p* = 0.0013), with a significant reduction in variance (*F* = 10.464, *p* = 0.0022) because higher decreases were observed in patients with higher baseline values (correlation between baseline values and decrease before/after treatment: Spearman *r* = 0.882, *p* < 0.0001) (Figure 1).

A GLM analysis showed that the pattern of change of IL-1β and IL1ra did not follow parallel slopes of time course, with IL-1β decreasing, and IL1ra increasing before/after treatment (interaction of analyte with time: *F* = 4.226; d.f. 1,50; *p* = 0.0450).

**FIGURE 2:**
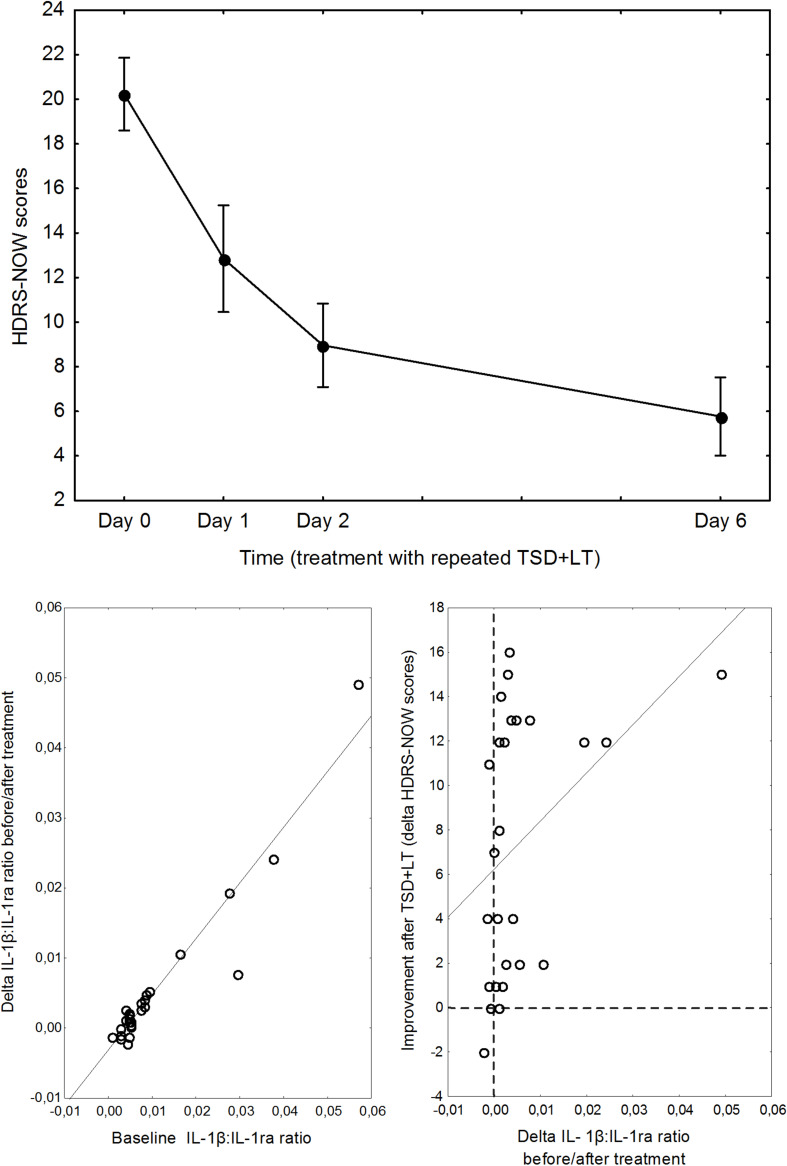
Top: Pattern of decrease of depression severity during the combined TSD+LT treatment. Points are means, whiskers are 95% confidence limits. Bottom, left: relationship between baseline IL1β:IL1ra ratio and its change before/after treatment. Bottom, right: relationship between delta IL1β:IL1ra ratio before/after treatment and improvement of depression.

Severity of depression significantly decreased after treatment, and the decrease in IL-1β:IL1ra ratio significantly influenced antidepressant response (Figure 2).

A repeated measures GLM ANOVA showed a marked improvement of depression severity over time (*F* = 69.37, d.f. 3,75, *p* < 0.0001), with the expected abrupt amelioration at the first TSD then followed by a further improvement, and a significant effect of the IL-1β:IL1ra ratio on the pattern of decrease of HDRS scores (interaction with time: *F* = 3.00, *p* = 0.0360).

Decomposition of the TSD cycles showed that the effect of IL-1β:IL1ra was significant on the improvement observed soon at the first cycle (*F* = 4.535, *p* = 0.0437). A GLZM analysis showed that the delta change in HDRS before/after TSD was significantly influenced by the IL-1β:IL1ra (LR χ^2^ = 4.500, *p* = 0.0339); results were significant also correcting for sex, age, and ongoing lithium treatment, which all lacked significant effects and could be excluded from the model according to the AIC.

## Discussion

The main finding of the present study is that antidepressant chronotherapeutic treatment with combined TSD+LT decreases the IL-1β:IL-1ra ratio in patients with bipolar depression, the decrease being proportional to the baseline IL-1β:IL-1ra ratio, and to the clinical efficacy of the TSD cycle: patients with higher baseline levels showed the higher decrease in IL-1β:IL-1ra, which associated with clinical response, while patients with lower baseline values showed negligible changes in the IL-1β:IL-1ra, and responded to treatment independent of them.

This observation supports the hypothesis that a reduction in the low-grade inflammation associated with major depression is part of the mechanism of action of TSD+LT, as suggested by previous clinical trials showing that effective antidepressant TSD activates gene expression pathways involved in cytokine signaling and immune function (Foo et al., 2019), and induces a robust increase of SCF (Benedetti et al., 2016a), which modulates microglial activity in the brain toward expressing anti-inflammatory cytokines (Terashima et al., 2018) and in particular downregulates the microglial expression of IL-1β in response to pro-inflammatory signaling (Zhang and Fedoroff, 1998). These specific effects could contribute to the observed reduction in IL-1β and increase in IL-1ra observed in our patients.

Moreover, our observation is in agreement with available clinical trials, which showed that only a fraction of depressed patients show higher levels of IL-1β and TNF-α hampering response to first-line antidepressants, and can then benefit of specific anti-inflammatory interventions (Raison et al., 2013; Benedetti et al., 2021). While higher IL-1β:IL-1ra ratio associated with the anti-inflammatory and clinical effects of treatment, at lower level response occurred rather independent of it, further supporting the complexity of the interplay between immune system and the treatment of depression, and the need of a personalized medicine approach to improve the efficacy of antidepressant treatments through immune modulation (Branchi et al., 2021).

In respect to TSD+LT, the anti-inflammatory component of its mechanism of action associates with multiple neurobiological effects, targeting brain monoamines, glutamate, and post-synaptic mechanisms (Benedetti and Smeraldi, 2009; Wirz-Justice and Benedetti, 2019). The reduction in IL-1β activity could contribute to the clinical antidepressant effects by reducing its detrimental effects on brain homeostasis and monoaminergic function: In animal models, IL-1β decreases hippocampal neurogenesis and induces depressive-like symptoms (Yoshimura et al., 2013; Yang et al., 2015); In depressed patients, it associates with decreased functional connectivity within the cortico-striatal reward circuitry (Gupta et al., 2016). Moreover, IL-1β can cause depression by upregulating indolamine-2,3-dioxygenase (IDO), thus activating the catabolism of tryptophan to kynurenine (Cattaneo et al., 2013), reducing brain serotonin (5-HT), and increasing the N-methyl-D-aspartate receptor agonist quinolinic acid (Belzeaux et al., 2012; Cattaneo et al., 2016); and by activating the HPA axis and inducing glucocorticoid receptors resistance (Powell et al., 2013; Guilloux et al., 2015). A reduction of IL-1β-mediated pro-inflammatory signaling is then expected to result in higher levels of brain 5-HT, reduced NMDA agonism, and increased neuroplasticity, all effects associated with antidepressant response.

Finally, IL-1β activity shows a bidirectional relationship with the circadian timing system. The biological clock controls IL-1β secretion in diverse tissues and immune cells, and when circadian rhythms are disrupted the inflammatory processes worsen in various medical conditions involving IL-1β signaling (Pourcet and Duez, 2020), also leading to behavioral consequences in animal models (Smith et al., 2021). On the other hand, IL-1β regulates sleep by acting on monoaminergic neurotransmission (Imeri and Opp, 2009) and specific neuronal receptors (Davis et al., 2015), and the disruption of the circadian rhythmicity of brain homeostatic mechanisms and inflammatory signaling has been proposed as a pathogenetic mechanism in mood disorders, which involve a marked disruption of all circadian mechanisms (Wirz-Justice and Benedetti, 2019). Combined TSD+LT acts on the biological clock and restores the cyclical patterns of brain homeostasis (Benedetti, 2012; Canali et al., 2014), and the reduction of IL-1β, which normally bridges innate and adaptive immunity, could be part of this general improvement in the synchronization of internal timing, possibly correcting circadian misalignment and abnormal relationships between homeostatic and circadian regulators of biological rhythms (Benedetti and Terman, 2013; McClung, 2013).

Strengths of the present study include a focused research question and a real-world experimental setting, but our results must be viewed in light of some limitations. The study is a before-after, proof-of-concept trial, which correlates changes in depressive symptomatology with changes in inflammatory biomarkers, and does not allow then to attribute them to the effect of the intervention. Further research including a proper control group from patients without TSD+LT intervention can address this limitation. Moreover, no patient was drug-naive, and the drug treatments administered during the course of the illness could have influenced the clinical and biological picture. Recruitment was in a single center and in a single ethnic group, thus raising the possibility of population stratifications. The sample was small, and the power was sufficient to detect a single effect in this proof-of-concept study, but not for studying interactions between biological and psychopathological factors. Individual chronotype of the patients, and the features of their sleep before TSD+LT could have influenced results. These limitations, however, do not bias the main finding of an effect of combined TSD+LT in reducing IL-1β:IL-1ra in bipolar depressed patients.

## Data Availability Statement

The original contributions presented in the study are included in the article/supplementary material, further inquiries can be directed to the corresponding author/s.

## Ethics Statement

The studies involving human participants were reviewed and approved by the Comitato Etico dell’IRCCS Ospedale San Raffaele, Milano. The patients/participants provided their written informed consent to participate in this study.

## Author Contributions

FB designed the study, analyzed the data, and wrote the first draft. SD, RZ, BB, and CC selected and treated the patients. EM collected and analyzed the data. CL performed all biological analyses. All authors critically revised the manuscript and take responsibility for data integrity.

## Conflict of Interest

The authors declare that the research was conducted in the absence of any commercial or financial relationships that could be construed as a potential conflict of interest.

## Publisher’s Note

All claims expressed in this article are solely those of the authors and do not necessarily represent those of their affiliated organizations, or those of the publisher, the editors and the reviewers. Any product that may be evaluated in this article, or claim that may be made by its manufacturer, is not guaranteed or endorsed by the publisher.
